# Comparative Evaluation of the Relationship Between Airway Inadequacy, Head Posture, and Craniofacial Morphology in Mouth-Breathing and Nasal-Breathing Patients: A Cephalometric Observational Study

**DOI:** 10.7759/cureus.47435

**Published:** 2023-10-21

**Authors:** Shashank Jaiswal, Faraz Sayed, Venkatesh V Kulkarni, Praveena Kulkarni, Pavan Tekale, Kapil Fafat

**Affiliations:** 1 Orthodontics, CSMSS (Chatrapati Shahuji Maharaj Shikshan Sanstha) Dental College and Hospital, Aurangabad, IND; 2 Orthodontics, Dr Faraz's Dental Clinic & Orthodontic Care, Pune, IND; 3 Oral Pathology and Microbiology, Bharati Vidyapeeth (Deemed to be University) Dental College and Hospital, Pune, IND; 4 Orthodontics, Dr. Rajesh Ramdasji Kambe Dental College and Hospital, Akola, IND

**Keywords:** craniofacial morphology, orthodontics, cephalometry, head-posture, air-way

## Abstract

Background: The process of respiration is the primary factor of the posture of the jaws and tongue. Thus, a changed respiratory form like mouth breathing can change the posture of the head, jaw, and tongue. This, in turn, could change the equilibrium of pressure on the jaws and teeth thus affecting jaw growth and teeth positions. The influence of nasorespiratory function on craniofacial growth has stimulated interest and debate for more than a century. Mouth breathing is the reason for numerous orthodontic glitches such as a mouth breather’s face evolving aberrantly because of of functional disruptions triggered by chronic airway obstruction. The relationship between nasorespiratory function and dentofacial development remains controversial despite the long-standing clinical concern of orthodontists, so there was a need to evaluate and compare the relationship between head posture, airway inadequacy, and craniofacial morphology in mouth breathers and nasal breathers.

Methodology: Forty patients were selected and divided equally into two groups: mouth breathers and nasal breathers. Patients were diagnosed as mouth breathers based on physical examination and a history of chronic allergic rhinitis, adenoid, and tonsil enlargement. Lateral cephalograms were taken for all patients in the natural head position (NHP) with the Planmeca Proline XC Dimax3 x-ray machine (Planmeca, Helsinki-Uusimaa, Finland). All lateral cephalograms were traced and analysis was done to check airway, head posture, and craniofacial morphology. Descriptive statistics were performed to obtain the means and standard deviation of all the sample sizes. Unpaired t test was performed between nasal breathers and mouth breathers to check and evaluate the relationship.

Result: Mouth-breathing patients varied from nasal-breathing patients in airway adequacy and craniofacial morphology. A little, but not statistically significant, difference was seen in head posture between the two groups.

Conclusions: Early interception of mouth breathing in patients could be very helpful, as the postural changes in the mouth-breathing patients, if continued for a longer period of time, could be the reason for severe skeletal deformities as well as dental malocclusion.

## Introduction

The relationship between nasorespiratory function and dentofacial development remains controversial despite the long-standing clinical concerns of orthodontists [1,2]. The influence of nasorespiratory function on craniofacial growth has stimulated interest and debate for more than a century [2]. This controversy began in the 19th century with subjective reports of clinicians who described a distinctive craniofacial morphology considered characteristic of “mouth-breathing” patients [3,4]. One of the first reports associating the habit of mouth breathing with deleterious physical and dental consequences was published by George Catlin in 1860 entitled “The Breath of Life Monograph” [2].

A normal mode of breathing is very important for a normal growth pattern. An abnormal breathing pattern can cause altered facial growth. It also causes changes in the human head posture. Children who are mouth breathers demonstrate more extension of the head related to the cervical spine, and condensed cervical lordosis, with more skeletal divergence, compared with nasal-breathing subjects [3-5].

A normal mode of breathing leads to optimal dentofacial growth. In addition to specific detrimental effects on the facial skeleton, impaired nasal breathing has been reported to cause changes in human head posture. Experimentally, a complete blockage of the nostrils causes an immediate elevation of the head [6-9].

Hence, there was a need to evaluate the relationship between altered facial growth and altered inclination of cervical vertebrae in mouth-breathing patients and distinguish them from nasal-breathing patients. This also aids in the diagnosis of mouth breathing by finding the cephalometric relation between cervical vertebrae inclination angle in mouth breathing and nasal breathing patients.

## Materials and methods

**Figure 1:**
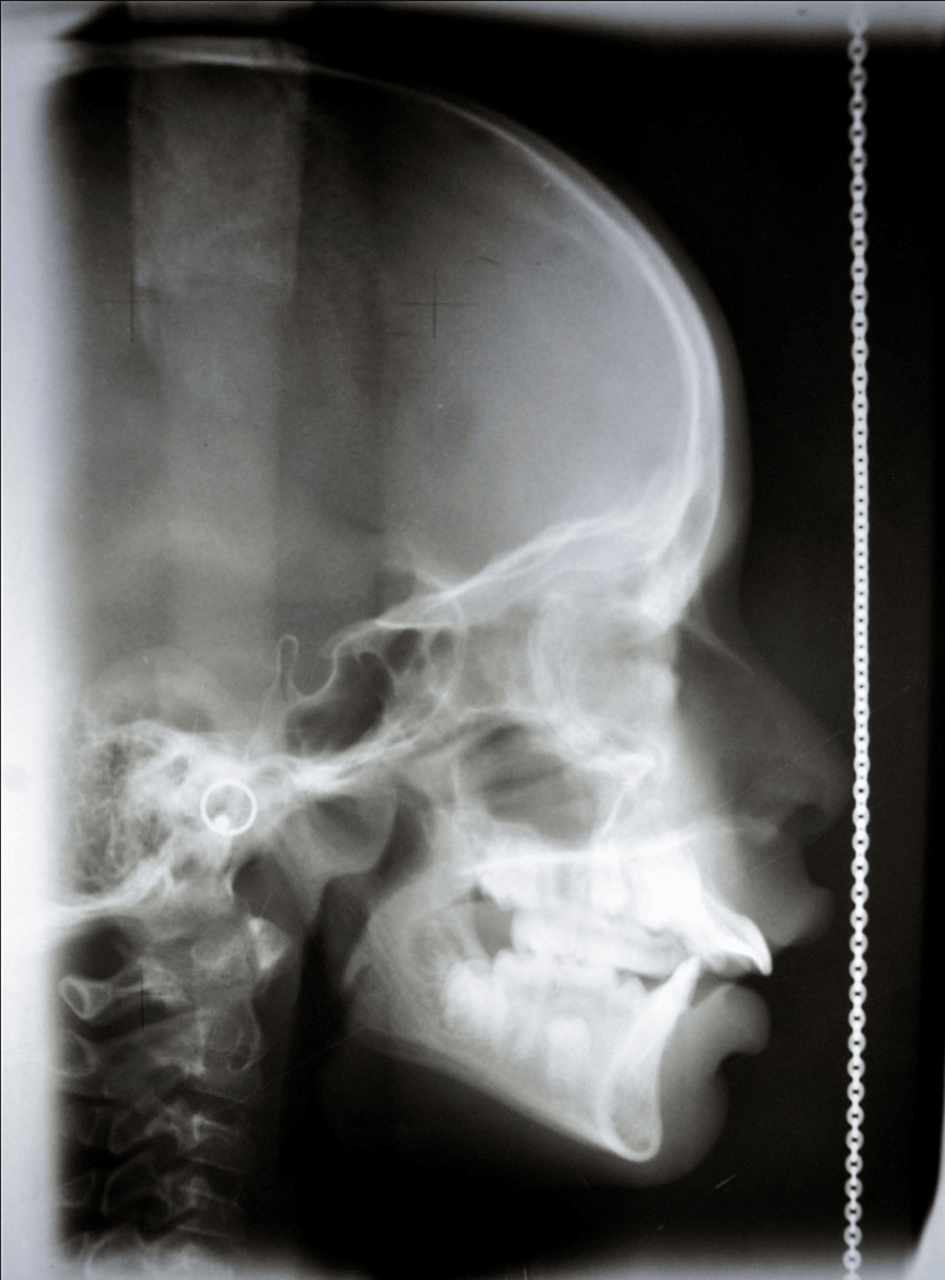
Lateral cephalogram of a patient

The study was conducted in the Department of Orthodontics, Dr. Rajesh Ramdasji Kambe Dental College and Hospital, Akola, Maharashtra, India. The total duration of the present study was two years. Further, the sample size comprises 40 patients (20 mouth breathers and 20 nasal breathers) in the age group of 9-25 years. Lateral cephalograms (Figure 1) were obtained from the Department of Oral Medicine Diagnosis and Radiology, Dr Rajesh Ramdasji Kambe Dental College and Hospital, Akola, Maharashtra, India.

The radiographic apparatus used was a Planmeca Proline XC Dimax3 X-ray machine (Planmeca, Helsinki-Uusimaa, Finland). The tube voltage was 80 KV, the current was 8 mA, and the exposure time was 0.8 seconds. The film-to-source distance was 5 feet 2 inches, and the distance between the film and the patient’s mid-sagittal plane was 6 feet.

Subjects were divided into two groups: Nasal breathers and Mouth breathers. The inclusion criteria for Nasal breathers were: Predominant nasal breathing with competent lips, no history of allergic rhinitis, adenoidal, tonsilar, or nasal septum deviation, and no history of orthodontic treatment or facial trauma. The inclusion criteria for Mouth breathers were: Predominantly mouth breathing with incompetent lips, a history of chronic allergic rhinitis, and adenoidal and tonsilar enlargement, no history of orthodontic treatment or facial trauma, nasal obstruction diagnosis by physical examination, radiograph, and opinion by an ENT surgeon, no history of cervical spondylitis, and no history of adenoid surgery.

**Figure 2:**
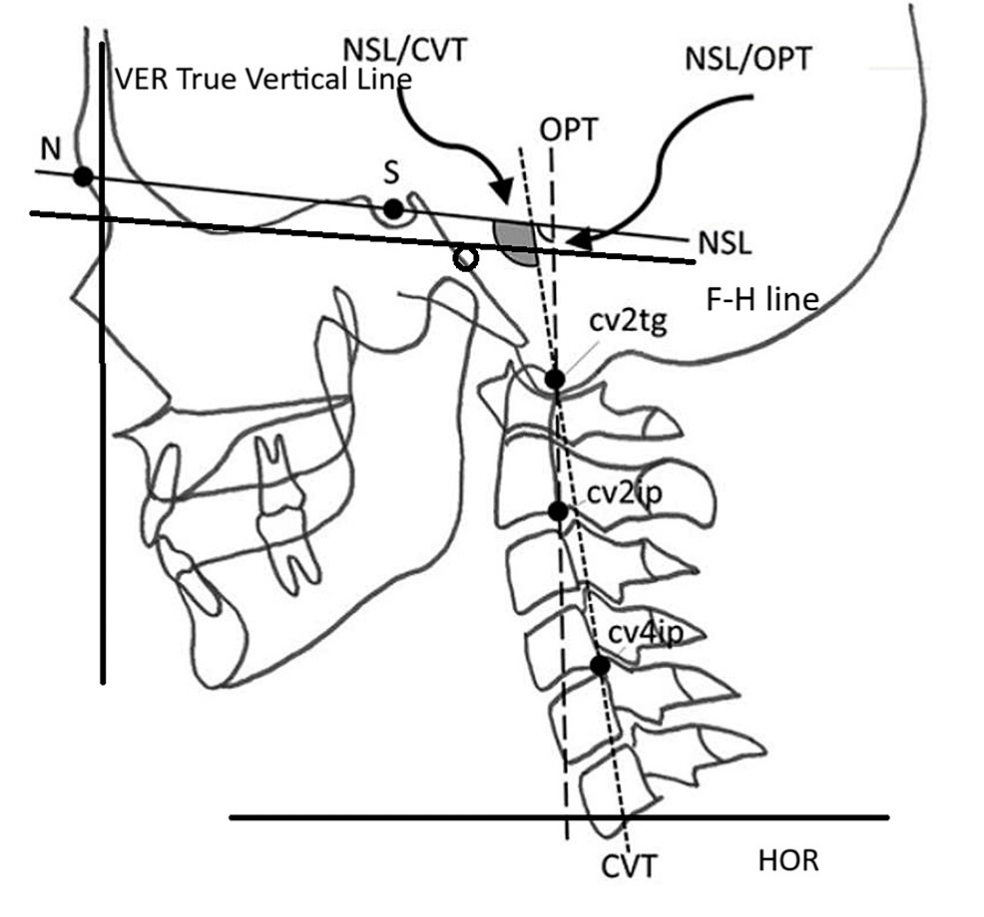
Craniovertical, craniocervical, and cervical inclination. NSL: Nasion-sella line; VER: True vertical line; FH: Frankfort line; OPT: Odontoid process tangent; CVT: Craniovertical; HOR: True horizontal line Image Source: Jacobson and Jacobson, 2006 [10]; used with permission from the publishers by the authors

All lateral cephalograms were traced and analyzed for further variables. The following craniovertical, craniocervical, and cervical inclination variables are to be determined: Craniovertical: nasion-sella line (NSL)/true vertical line (VER) (°), Frankfort line (FH)/VER(°); Craniocervical: NSL/odontoid process tangent (OPT)(°), NSL/craniovertical (CVT)(°), FH/OPT(°), FH/CVT(°); Cervical inclination: OPT/true horizontal line (HOR)(°), CVT/HOR(°) (Figure 2). CVT is the line through the most postero-inferior point on the corpus of the second and fourth cervical vertebra.

**Figure 3:**
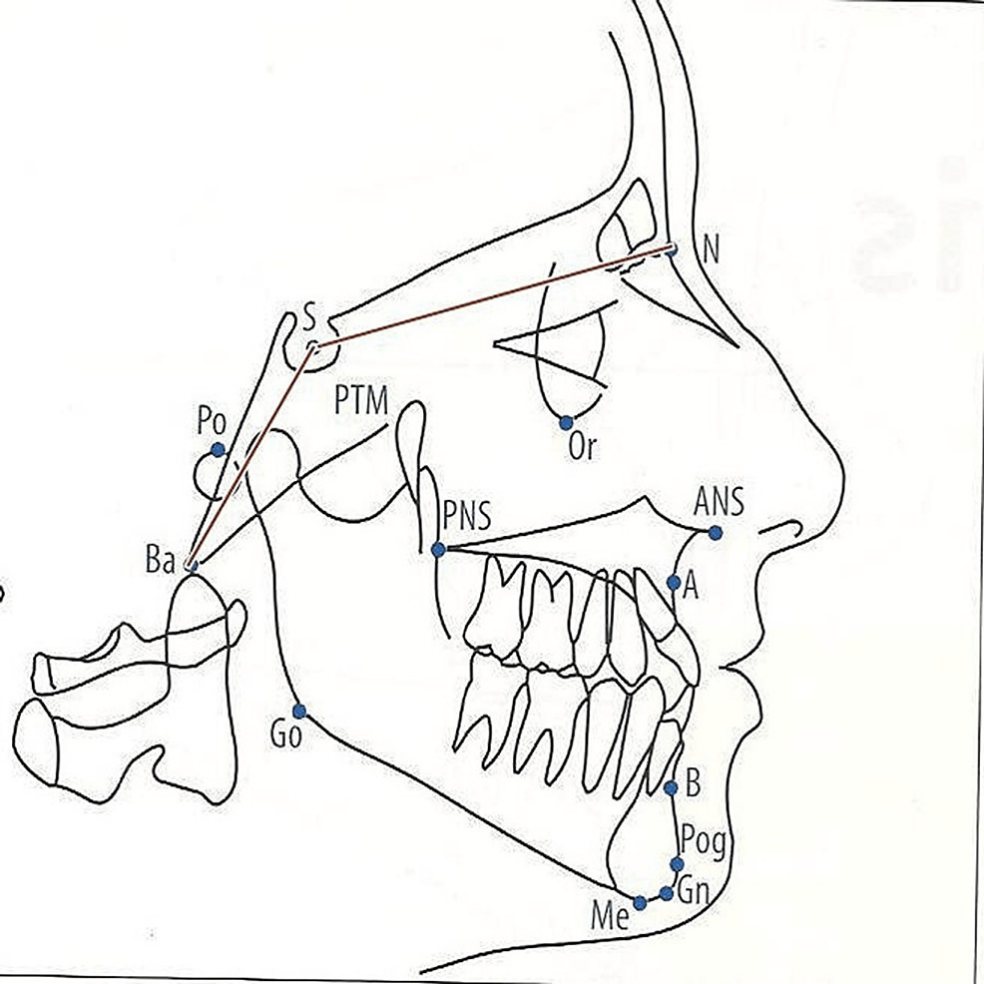
Measurement of cranial base variables ANS: Anterior nasal spine; PNS: Posterior nasal spine; Ba: Basion; Bo: Bolton point; Go: Gonion;  Gn: Gnathion; Me: Menton; N: Nasion; Or: Orbitale; Pog: Pogonion; Po: Porion; Point A: Subspinale; Point B: Supramentale; PTM: Pterygomaxillare; S: Sella Image Source: Jacobson and Jacobson, 2006 [10]; used with permission from the publishers by the authors

Cervical inclination about the true horizontal was expressed by variables, OPT/HOR and CVT/HOR. The cervical inclination angles are important in contributing to large changes in the craniocervical relationship. These angles usually show the same relationship to craniofacial development as the craniocervical angles but with the opposite sign, due to the construction of the angle. Craniofacial morphology measurements include cranial base variables such as the nasion (N)-sella (S) line (NSL) (mm), S-basion (Ba) line (S-Ba) (mm), and N-S-Ba(°) as shown in Figure 3.

**Figure 4:**
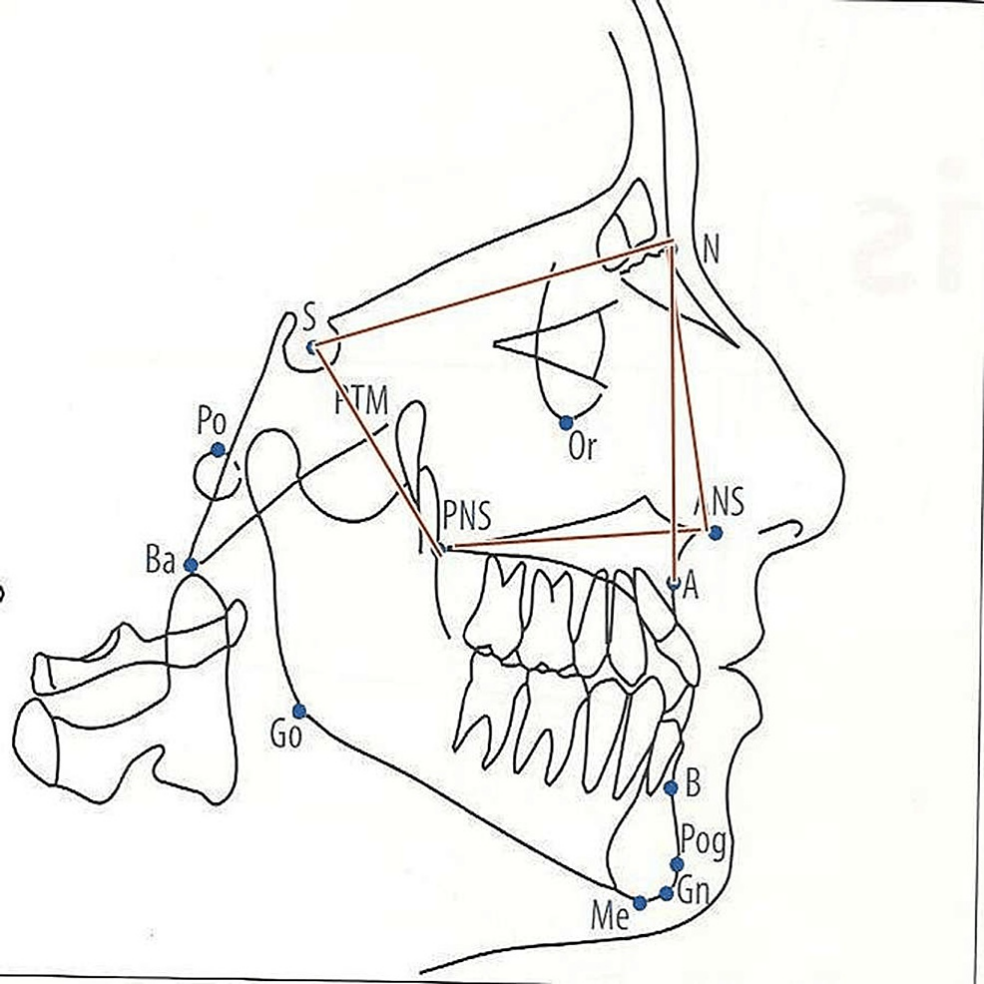
Maxillary arch variables ANS: Anterior nasal spine; PNS: Posterior nasal spine, N: Nasion; S: Sella; SNA: Sella Nasion Point A; Ba: Basion; Go: Gonion;  Gn: Gnathion; Me: Menton; N: Nasion; Or: Orbitale; Pog: Pogonion; Po: Porion; Point A: Subspinale; Point B: Supramentale; PTM: Pterygomaxillare; S: Sella Image Source: Jacobson and Jacobson, 2006 [10]; used with permission from the publishers by the authors

**Figure 5:**
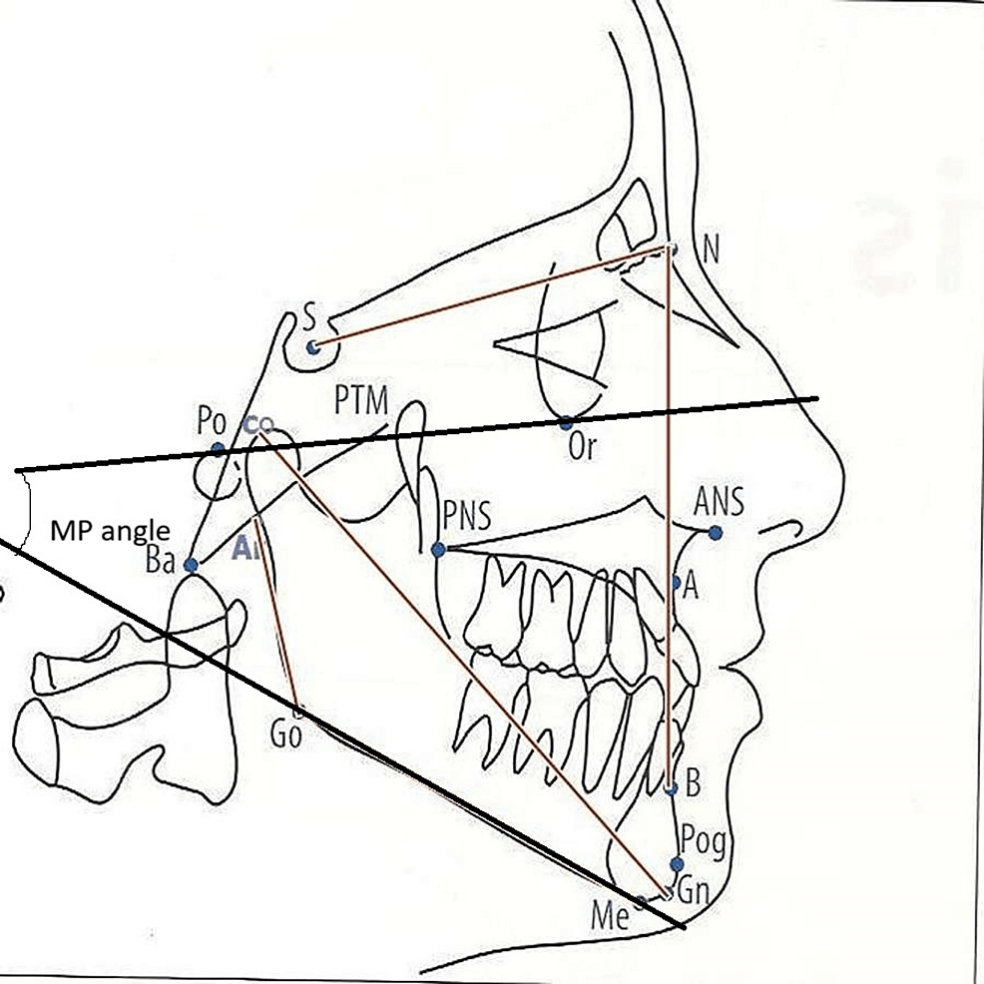
Mandibular variables Co-Gn: Condylion-Gnathion; SNB angle: Sella-Nasion-Point B angle, RL: Ramus line; ML: Mandibular line; ANS: Anterior nasal spine; PNS: Posterior nasal spine, N: Nasion; S: Sella; Ba: Basion; Go: Gonion;  Me: Menton; N: Nasion; Or: Orbitale; Pog: Pogonion; Po: Porion; Point A: Subspinale; Point B: Supramentale; PTM: Pterygomaxillare; S: Sella Image Source: Jacobson and Jacobson, 2006 [10]; used with permission from the publishers by the authors

The maxillary arch and the mandibular arch variables with respect to the cranial base were measured as shown in Figure 4 and Figure 5, respectively. Figure 4 shows the evaluation of maxillary parameters such as anterior nasal spine (ANS)-posterior nasal spine (PNS), N-ANS, S-PNS, N perpendicular to point A, and angle S-N point A (SNA). Mandibular variables evaluated were Condylion-Gnathion (Co-Gn), N perpendicular to point pogonion (Pog), SNB angle, gonial angle, and mandibular plane angle as shown in Figure 5. Gonial angle refers to the angle that is formed by the ramus line (RL), and the mandibular line (ML), where RL is the tangent to the posterior border of the mandible and ML is the lower border of the mandible through the gnathion and mandibular plane angle.

**Figure 6:**
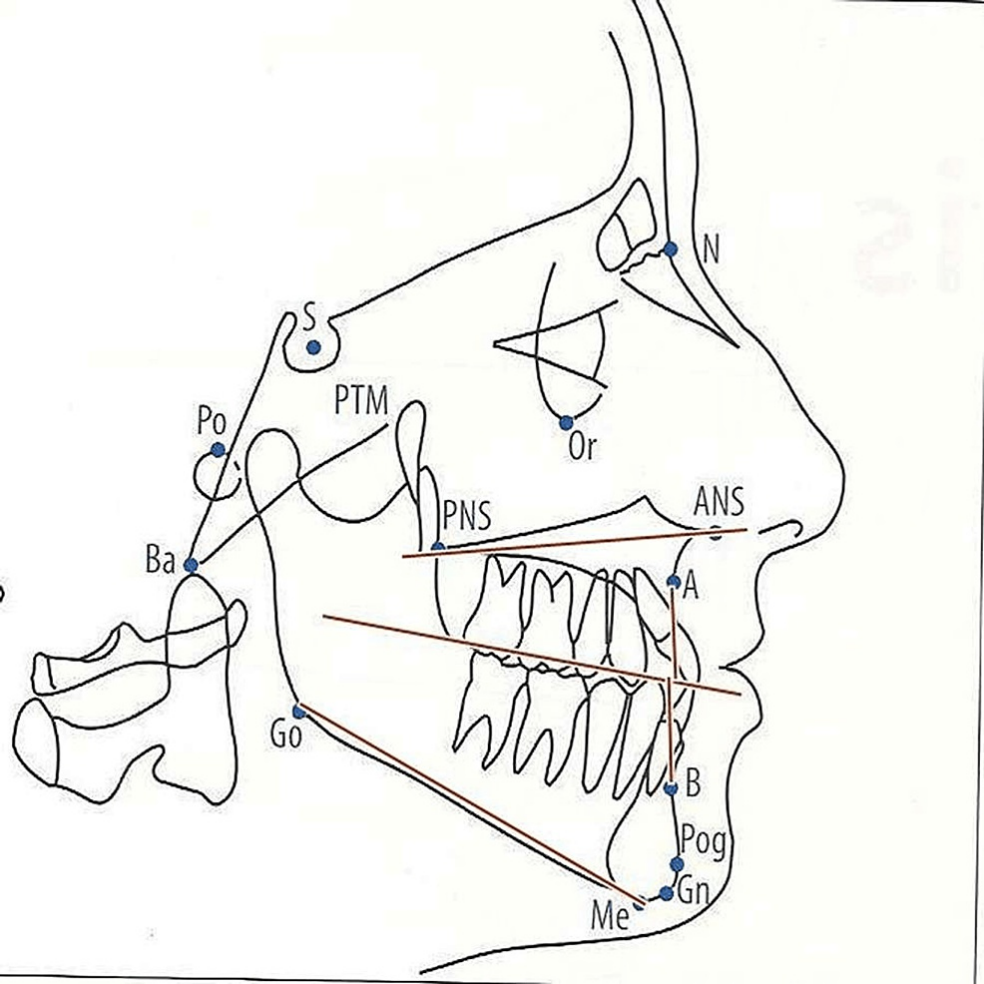
The jaw relation variables The jaw relation variables measure the palatal-mandibular plane angle (ANS-PNS/Go Me). Gn: Gnathion; ANS: Anterior nasal spine; PNS: Posterior nasal spine, N: Nasion; S: Sella; Ba: Basion; Go: Gonion;  Me: Menton; N: Nasion; Or: Orbitale; Pog: Pogonion; Po: Porion; Point A: Subspinale; Point B: Supramentale; PTM: Pterygomaxillare; S: Sella Image Source: Jacobson and Jacobson, 2006 [10]; used with permission from the publishers by the authors

Jaw Relation variables (Figure 6) such as the palatal-mandibular plane angle (ANS-PNS/Go-Me) are to be measured. 

**Figure 7:**
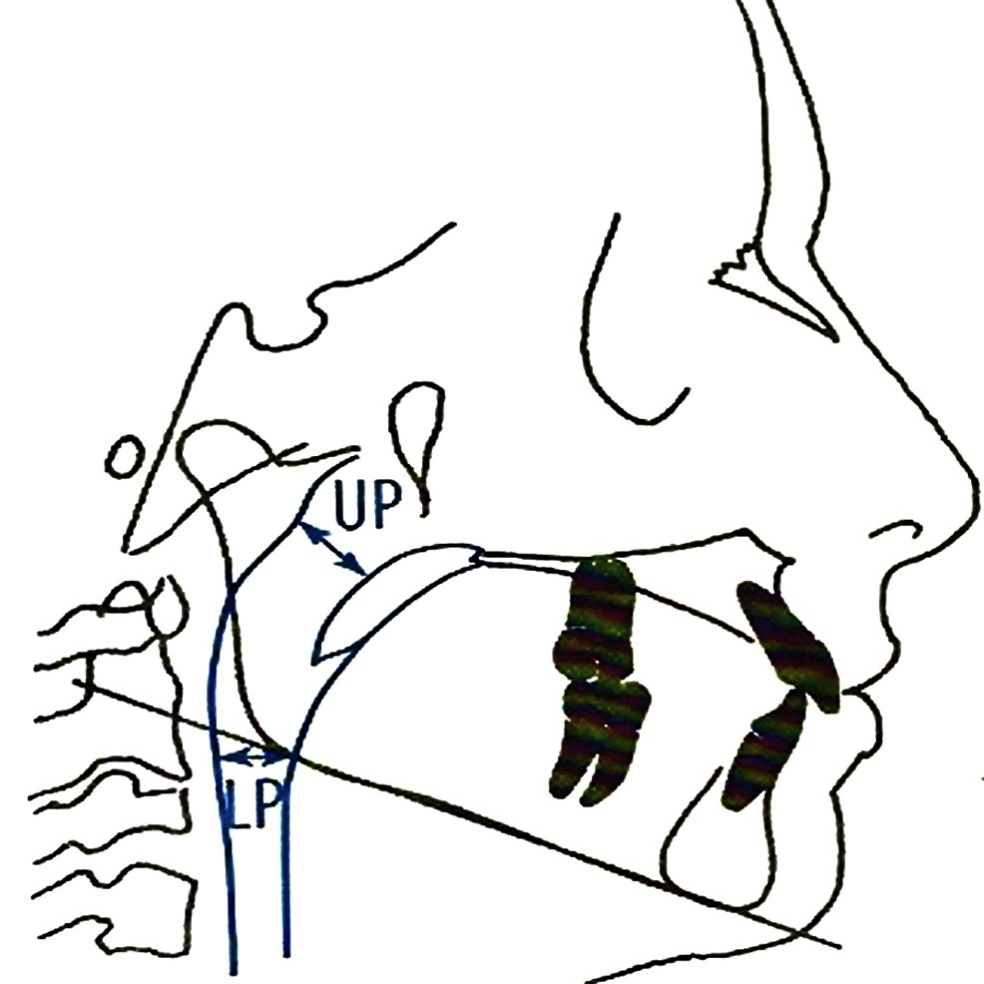
The airway variables The airway variables determine the width of the upper pharynx (UP) and lower pharynx (LP) as suggested by McNamara [11] Image Source: Jacobson and Jacobson, 2006 [10]; used with permission from the publishers by the authors

The upper and lower airway variables are to be measured as shown in Figure 7. Upper pharyngeal width is calculated from a point on the posterior outline of the soft palate to the nearest point on the pharyngeal wall. This dimension is taken on the anterior half of the soft palate outline. Lower pharyngeal width is calculated from the point of intersection of the posterior border of the tongue and the inferior border of the mandible to the nearest point on the posterior pharyngeal wall.

## Results

Descriptive statistics were performed to obtain means and standard deviations of the sample size. Unpaired t test was performed to evaluate and compare the head posture in mouth-breathers and nasal-breathers and results are shown in Table 1.

**Table 1:** Head posture in nasal-breathing patients and mouth-breathing patients. NSL: Nasion-sella line; VER: True vertical line; FH: Frankfort line; OPT: Odontoid process tangent; CVT: Craniovertical; HOR: True horizontal line

Head posture parameters	Mouth breathers (n=20)	Nasal breathers (n=20)	P value
Craniovertical			
NSL/VER(°)	103.25 ± 3.059	98.70 ± 23.045	0.387
FH/VER(°)	95.65 ± 4.056	96.15 ± 4.069	0.699
Craniocervical			
NSL/OPT(°)	100.40 ± 9.439	99.25 ± 7.732	0.676
NSL/CVT(°)	102.75 ± 7.217	103.50 ± 9.811	0.785
FH/OPT(°)	95.80 ± 12.190	93.25 ± 8.765	0.452
FH/CVT(°)	102.45 ± 21.860	97.05 ±8.678	0.311
Cervical inclination			
OPT/HOR(°)	91.95 ± 9.242	93.60 ± 8.768	0.566
CVT/HOR(°)	87.30 ± 8.374	90.70 ± 8.342	0.206

Unpaired t test was performed to evaluate and compare the facial morphology in mouth-breathing and nasal-breathing patients and results are shown in Table 2.

**Table 2:** Comparison of facial morphology in mouth-breathing and nasal-breathing patients. *Statistically significant, unpaired t test (P<0.05) N: Nasion; S: Sella; Ba: Basion; ANS: Anterior nasal spine; PNS: Posterior nasal spine; Point A: Subspinale; SNA: Sella-nasion-point A angle; Co-Gn: Condylion-Gnathion; Pog: Pogonion; Go: Gonion; Me: Menton; O: Oribitale; Ar: Articulare

Variables	Mouth Breathers (n=20), mean±SD	Nasal Breathers (n=20), mean±SD	P-Value
Cranial Base			
N-S (mm)	70.45±4.032^*^	74.25±3.416	0.003
S-Ba (mm)	45.65±2.978	46.45±3.252	0.422
N-S-Ba(°)	130.05±4.045	132.55±4.785	0.082
Maxilla			
ANS-PNS (mm)	51.50±2.743^*^	55.20±4.150	0.002
N-ANS (mm)	51.35±8.106	54.75±9.369	0.227
S-PNS (mm)	46.65±4.344	49.10±3.698	0.062
N perpendicular to point A (mm)	-3.30±4.118	-1.25±3.959	0.117
SNA(°)	80.50±3.332^*^	82.85±3.528	0.014
Mandible			
Co-Gn (mm)	105.60±5.500^*^	113.25±6.920	0.000
N perpendicular to point Pog (mm)	-10.40±7.749	-9.95±6.700	0.845
SNB(°)	74.15±9.815	78.35±3.588	0.085
Ar-Go-Me angle(°)	130.40±6.378^*^	125.10±5.220	0.007
NS-Mandibular plane (Go-Me)(°)	28.70±4.105	26.90±7.055	0.330
Facial height			
N-Me	114.30±5.536	118.90±9.397	0.067
ANS-Me	63.40±11.927	68.25±8.663	0.149
S-Go	74.34±8.035^*^	79.60±6.012	0.025
Jaw Relation			
ANB(°)	3.40±3.440	4.45±2.564	0.281
BO-AO (mm)	3.800±3.6935	2.875±4.2977	0.470
Palatal-Mandibular plane(°)	31.10±5.108	28.55±5.826	0.012

Unpaired t test was performed to evaluate and compare the airway in mouth-breathing patients and nasal-breathing patients and results are shown in Table 3.

**Table 3:** Comparison of the airway in mouth-breathing and nasal-breathing patients. *Statistically significant, unpaired t test (P<0.05)

Airway	Mouth Breathers, mean±SD	Nasal Breathers, mean±SD	P-Value
Upper airway	7.60 ± 3.662	16.05 ± 3.605	0.000
Lower airway	8.70 ± 2.179	12.40 ± 1.875	0.000

Craniocervical postural variables

There is an increase in NSL/OPT, NSL/CVT, FH/OPT, and FH/CVT parameters in the mouth-breathing patients as compared to the nasal breathing patients but this was not statistically significant, as shown in Table 1. This indicates that there is an upward and backward rotation of the head and a cervical extension.

Craniovertical postural variables

There is a slight increase in NSL/VER and FH/VER parameters in the mouth-breathing patients as compared to the nasal-breathing patients but it was not statistically significant, as shown in Table 1. This indicates that there is a mild change in the head posture in mouth-breathing patients attributed to the upward and backward tilting of the head. 

Cervical vertebrae inclination

Cervical inclination about the true horizontal was expressed by variables, OPT/HOR and CVT/HOR. The cervical inclination angles are important in contributing to large changes in the craniocervical relationship. These angles usually show the same relationship to craniofacial development as the craniocervical angles but with the opposite sign, due to the construction of the angle.

Craniofacial morphology

Craniofacial morphology includes maxillary variables and mandibular variables with respect to cranial base and jaw relation. There was a statistically significant difference in SNA angle (p=0.014). Similarly, the length of the maxilla (ANS-PNS) was reduced in mouth-breathing patients, which shows a statistically significant difference, as shown in Table 2. There was a statistically significant difference in gonial angle (Ar-Go-Me)(p=0.007).

There was a statistically significant difference in the palatal-mandibular plane angle (ANS-PNS/Go-Me). The result of our study shows a statistically significant difference in the anterior cranial base length (S-N). Mouth-breathing patients also showed differences in head posture when compared to nasal-breathing patients, but it was not statistically significant. While comparing facial morphology in both groups, mouth-breathing patients showed statistical differences in craniofacial morphology.

Thus, overall mouth-breathing patients varied from nasal-breathing patients in airway adequacy and craniofacial morphology and a little, but not statistically significant, difference in head posture as shown in Table 3.

## Discussion

Respiratory needs are the essential determinants of the pose of the jaws and tongue (and of the head itself, to a lesser degree) [1-3]. Subsequently, a changed respiratory design, such as breathing through the mouth instead of the nose, may alter the pose of the head, jaw, and tongue. Verbal breath modifies the muscle powers applied by the cheeks, tongue, and lips upon the maxillary arch, driving to a contract maxillary arch with a high palatal vault, a posterior crossbite, an anterior open bite, and a Class II or III dental malocclusion.

In the present study, the selection criteria of mouth breathers were distinctively different from the nasal breathing pattern patient. McNamara (1984) checked the upper and lower pharyngeal width [11]. Based on his study, we checked the width of upper and lower pharyngeal spaces on cephalograms. A clinical examination was carried out to check lip incompetency and tonsillar and adenoid enlargement, and a simple water test and mirror test were performed on all the samples to evaluate breathing patterns. Based on all these criteria, the sample was divided into two groups: mouth-breathing and nasal-breathing patients. Patients who had shown positive correlation for all tests were categorized as mouth breathers and patients who showed negative correlation were grouped into nasal breathing patterns [2-5].

The result of our study showed that there is a slight increase in NSL/VER and FH/VER parameters in the mouth-breathing patients as compared to the nasal-breathing patients, but it was not statistically significant. This indicates that there is a mild change in the head posture in mouth-breathing patients attributed to the upward and backward tilting of the head. Shrivastava and Thomas [8] and Cuccia et al. [7] had taken similar parameters to check head posture. They showed a statistically significant difference in craniocervical angles. The result of our study shows a statistically significant difference in SNA angle. This angle is reduced in mouth-breathing patients when compared with nasal-breathing patients. This angle indicates the relative anteroposterior positioning of the maxilla in relation to the cranial base. Therefore, the reduced angle indicates a relative backward retrusion of the maxilla about the cranial base. Similarly, the length of the maxilla (ANS-PNS) was reduced in mouth-breathing patients, which shows a statistically significant difference. The result of our study shows a statistically significant difference in gonial angle (Ar-Go-Me). This angle is increased in mouth-breathing patients when compared to nasal-breathing patients. The increased angle indicates the downward and backward rotation of the mandible. Similarly, mandibular plane angle (NS-GoMe) is increased in mouth breather patients as compared to nasal breather patients but is not statistically significant. Both parameters, Ar-Go-Me and NS-GoMe, show that there is a tendency towards vertical growth in mouth breathers. These findings were similar to the study done by Shrivastava and Thomas [8] and Solow et al. [3].

Cervical inclination about the true horizontal was expressed by variables, OPT/HOR and CVT/HOR. The cervical inclination angles are important in contributing to large changes in the craniocervical relationship. These angles usually show the same relationship to craniofacial development as the craniocervical angles but with the opposite sign, due to the construction of the angle. Huggare and Laine-Alava, in 1997, assessed the relationship between nasorespiratory function and parameters of head posture using 58 young adults, aged 19-33 years [12]. The outcome displays enlarged craniocervical angulation and forward inclination of the cervical spine in subjects with a relatively large nasal cross-sectional area, but this study also confirms that there is an upsurge in head extension in obstructed subjects.

The jaw relation variable result of the current study showed a statistically significant difference in the palatal-mandibular plane angle (ANS-PNS/Go Me). This angle was increased in mouth-breathing patients when compared with the nasal-breathing patients. The increased angle indicates the downward and backward rotation of the mandible. These findings were similar to the study done by Shrivastava and Thomas [8], Cuccia et al. [7], and Solow et al. [3]. The result of our study shows a statistically significant difference in the anterior cranial base length (S-N). The length of the anterior cranial base was reduced in mouth-breathing patients as compared to the nasal breathing patients. Shrivastava and Thomas showed no significant variation in mouth breather and nasal breather patients [8]. Solow et al. [3] and Huggare and Laine-Alava [12] demonstrated airway adequacy by rhinomanometric tests. In the present study, we evaluated the width of the upper and lower pharynx as suggested by McNamara [11]. This width was then compared between nasal and mouth-breathing patients.

The result of our study showed that there is a statistically significant difference in airway adequacy in mouth-breathing patients. The upper and lower pharyngeal width was decreased in mouth breathers. The decreased pharyngeal width could be due to faulty positioning of the tongue, retrognathism of the mandible, which decreases the volume of the orofacial capsule, or due to tonsillar or adenoid enlargement.

The concept of natural head position (NHP) was introduced to orthodontics in the 1950s. All the lateral cephalograms were taken in NHP as it is easily reproducible and is a standard head posture. The radiographs were exposed in the NHP keeping the mirror in front with the subjects standing in ortho position and focusing into their eyes and lips in repose with maximum intercuspation of the occlusion [12-15]. The VER was indicated on the films with a 0.5 mm weighted lead wire mounted on the head holder. The concept of NHP is not new as it has been studied by various authors [3,16-19]. They all have advocated the use of HOR as a base reference plane in the cephalometric analysis.

Solow et al. did a study in 1984 to see if there is a connection between the size of the upper airway and the angle between the head and neck using cephalometric techniques [3]. They found that when the space in the nasopharynx is reduced, the angle between the head and neck increases. However, in the present study, we found that mouth-breathing patients with a smaller upper airway width did not have a significant difference in the angle between the head and neck compared to patients who are nasal breathers.

Breathing through the mouth during growth and development can affect NHP as well as craniofacial morphology. When teenagers begin breathing through their nose instead of their mouths, it could help their faces and skulls grow in a more normal way. When people breathe through their mouths, they tend to lift their heads higher and stretch their necks more.

Limitations of the present study

A larger sample size evaluation is needed. In the present study, limited sample sizes were evaluated. Instead of lateral cephalogram, the use of CT would have given more accurate images which would have helped for more accurate evaluation of parameters. Further clinical studies are necessary to evaluate this parameter with larger samples and methodological refinements. Longitudinal studies are needed to evaluate the effectiveness of early intervention in preventing these growth alterations. This further stresses the role of general dental practitioners, pedodontists, and orthodontists to identify the mouth breathing habit at the earliest possible age to prevent the impending changes associated with it.

## Conclusions

There is a difference in head posture in mouth breathers as compared with nasal breathers, which is statistically significant. Furthermore, there is a definite change in craniofacial morphology in mouth-breathing patients as compared to nasal-breathing patients. Mouth breather patients are generally associated with skeletal Class II pattern, vertical growth pattern, decreased anterior cranial base length, and decreased effective maxillary and mandibular lengths.

Our results showed airway inadequacy in mouth-breathing patients. This study gives cephalometric measurements, which can aid in the diagnosis of mouth-breathing patterns and early interception of mouth-breathing patterns in patients. This could be very helpful in the normalization of growth and maintainance of head posture, airway adequacy, and craniofacial morphology.
